# Incorporation of Personal Single Nucleotide Polymorphism (SNP) Data into a National Level Electronic Health Record for Disease Risk Assessment, Part 1: An Overview of Requirements

**DOI:** 10.2196/medinform.3169

**Published:** 2014-07-24

**Authors:** Timur Beyan, Yeşim Aydın Son

**Affiliations:** ^1^Informatics InstituteDepartment of Health InformaticsMiddle East Technical UniversityAnkaraTurkey

**Keywords:** health information systems, clinical decision support systems, disease risk model, electronic health record, epigenetics, personalized medicine, single nucleotide polymorphism

## Abstract

**Background:**

Personalized medicine approaches provide opportunities for predictive and preventive medicine. Using genomic, clinical, environmental, and behavioral data, tracking and management of individual wellness is possible. A prolific way to carry this personalized approach into routine practices can be accomplished by integrating clinical interpretations of genomic variations into electronic medical records (EMRs)/electronic health records (EHRs). Today, various central EHR infrastructures have been constituted in many countries of the world including Turkey.

**Objective:**

The objective of this study was to concentrate on incorporating the personal single nucleotide polymorphism (SNP) data into the National Health Information System of Turkey (NHIS-T) for disease risk assessment, and evaluate the performance of various predictive models for prostate cancer cases. We present our work as a miniseries containing three parts: (1) an overview of requirements, (2) the incorporation of SNP into the NHIS-T, and (3) an evaluation of SNP incorporated NHIS-T for prostate cancer.

**Methods:**

For the first article of this miniseries, the scientific literature is reviewed and the requirements of SNP data integration into EMRs/EHRs are extracted and presented.

**Results:**

In the literature, basic requirements of genomic-enabled EMRs/EHRs are listed as incorporating genotype data and its clinical interpretation into EMRs/EHRs, developing accurate and accessible clinicogenomic interpretation resources (knowledge bases), interpreting and reinterpreting of variant data, and immersing of clinicogenomic information into the medical decision processes. In this section, we have analyzed these requirements under the subtitles of terminology standards, interoperability standards, clinicogenomic knowledge bases, defining clinical significance, and clinicogenomic decision support.

**Conclusions:**

In order to integrate structured genotype and phenotype data into any system, there is a need to determine data components, terminology standards, and identifiers of clinicogenomic information. Also, we need to determine interoperability standards to share information between different information systems of stakeholders, and develop decision support capability to interpret genomic variations based on the knowledge bases via different assessment approaches.

## Introduction

The digital age is revolutionizing the old and historical population-based health care paradigm toward personalized medicine. Traditional medical approaches are not sufficiently predictive and preventive, as they focus on the manifestation of symptoms that often hide risk factors. Determining risk factors allows for prevention through early diagnosis, and provides new opportunities for developing personalized medicine approaches based on patient-centered, predictive, preventive, and effective health care services [1].

Genomic data and its derivatives (transcriptomes, proteomes, metabolomes, etc) are the essential elements of personalized medicine [2,3]. Every individual has almost four million variations in their own genome, when compared to the reference sequence. Genomic variations can range from single nucleotide changes to the gain or loss of whole chromosomes. Single nucleotide polymorphisms (SNPs), where a single nucleotide in the genome alters between individual or paired chromosomes, are about 90% of genomic variants, and some are already validated as important markers in the clinical practice, while others are on the way [4-6].

The rapid developments in next generation sequencing (NGS) technologies have substantially reduced both the cost and the time required to sequence the entire human genome, and it is expected that NGS-based analyses, for example, whole genome sequencing (WGS) and whole exome sequencing (WES), will be available for routine use in health care and prevention of disease by 2020 [7]. Providing genomic data to medical professionals will facilitate clinical decisions based on the individual’s genome, and allow tailoring health care services to the patient’s specific needs and characteristics [8]. In parallel, direct-to-consumer (DTC) genome-wide profiling tests are being developed to assess individual disease risks for many common polygenic diseases [9]. DTC genomic companies, for example, 23andMe, GenePlanet, and DNA DTC generally perform a gene-chip analysis of SNPs using deoxyribonucleic acid (DNA) extracted from saliva or serum sample [10-12].

In clinical decision processes, genomic variant data can be used for assessing disease risks, predicting susceptibility, early clinical diagnosing, following the course of the disease, targeted screening, and planning treatment regimens [3,13]. A reasonable way to carry this personalized approach into routine for medical practices would be integrating genotype data and its clinical interpretation within the electronic medical records (EMRs)/electronic health records (EHRs) [8,14].

Today, in many developed and developing countries, use of EMRs/EHRs is inevitable for health care providers for reimbursement of services, and to track the quality of the health care provided [15,16]. Recently, several EHR networks have been constituted in many countries of the world, including the National Health Information System of Turkey (NHIS-T) [17]. These EHR systems and networks have high potential for integrating genomic data in health care practices for personalized medicine.

In this work, as an initial attempt to develop a sophisticated infrastructure, we focused to incorporate the personal SNP data into NHIS-T for disease risk assessment, and evaluated the performance of various predictive models for prostate cancer cases. We presented our work as three parts: (1) a literature review for requirements, (2) the incorporation of SNP into the NHIS-T [18], and (3) an evaluation of SNP incorporated into NHIS-T for prostate cancer [19]. In this part, the scientific literature was reviewed, and the requirements were extracted regarding SNP data integrated EMRs/EHRs.

## Methods

The informatics pipeline for genome sequencing can be divided into several analytical steps, for example, base calling, alignment, variant analysis, interpretation, and in all levels different file formats are generated [20-22]. Currently, tools and techniques are developed for automated and reliable analysis, but clinical interpretation of variant data is still a major problem [21].

Today, most of the EMRs/EHRs are designed to store and retrieve the laboratory values and clinical findings, but do not have the ability to manage genomic data [23-25]. After WGS/WES, a file that contains a large number of variant data is acquired [26]. An entire genome sequence (the size of the haploid human genome) contains about 3 billion base pairs, and a single WGS data file is about 3 gigabytes. Storing and sharing of personal raw genomic sequences exceeds the transmission and storage capacity in many health care organizations [27]. Due to these technical limitations, raw genomic data are generally stored outside of the EMR; similar to picture archiving and communication systems for medical images, and clinical interpretation of the genomic data is preferably sent to the database of the EMR [28-30].

The initiatives of integrating a patient's genomic data into EMRs/EHRs is of a preliminary nature [31], and, until recently, only a few successful systems have been established, such as Cerner’s Genomics Solutions, McKesson’s Horizon Clinicals, and GeneInsight [26,32].

In the literature, basic requirements of genomic-enabled EMRs/EHRs are listed as incorporating genotype data and its clinical interpretation into EMRs/EHRs, developing accurate and accessible clinicogenomic interpretation resources (knowledge base), the interpretation and reinterpretation of variant data, and the immersion of clinicogenomic information into the medical decision processes.

**Figure 1:**
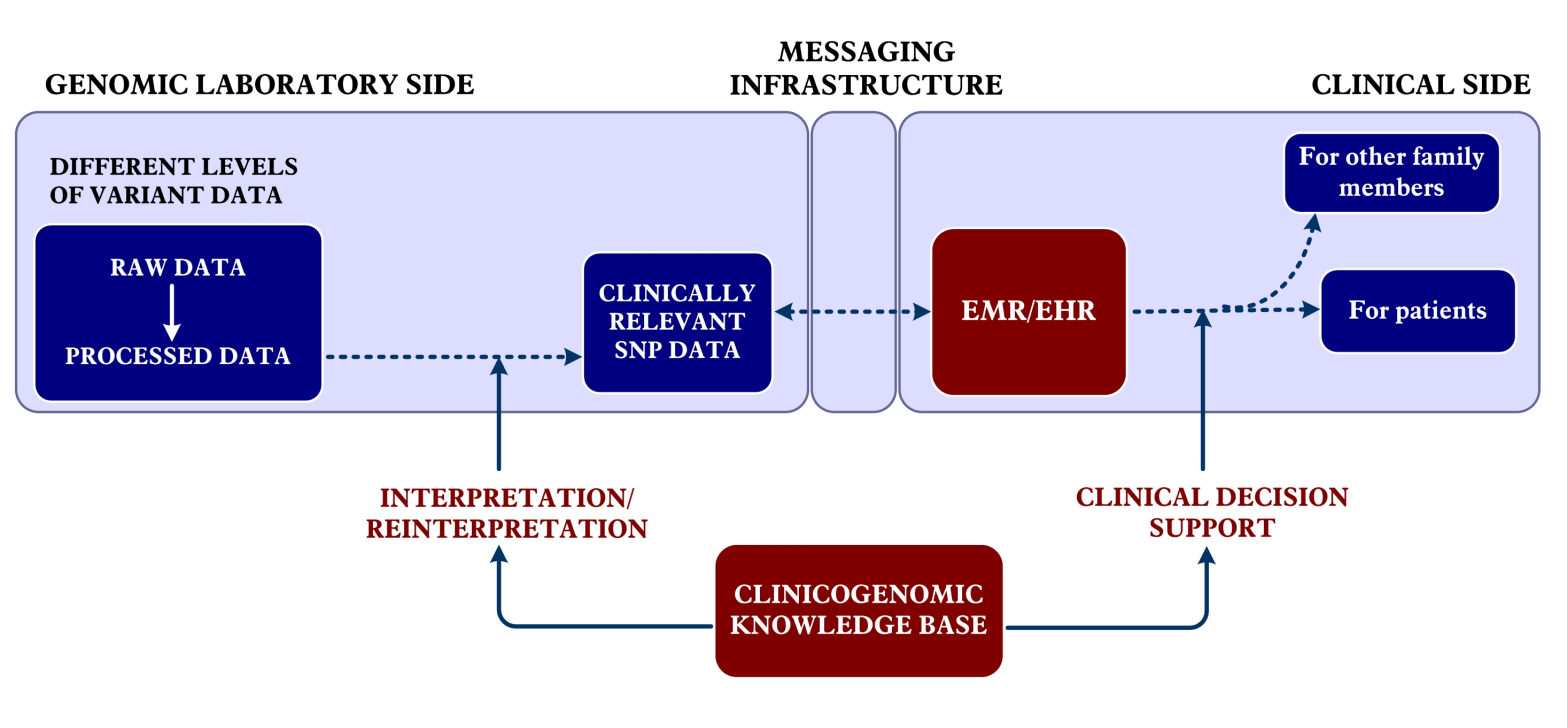
Main components of a genome-enabled electronic medical record/electronic health record. SNP; single nucleotide polymorphisms.

Figure 1 shows, in the genome laboratory side, various levels of sequence data can be produced. Since clinicians need an actionable clinical interpretation of the variant data, it is sufficient to share clinically relevant data between the laboratory and the clinical systems. The development of a clinicogenomic knowledge base is an obligation to extract clinical meaning from the variant data. On the clinical side, it is necessary to use decision support systems due to the high number of variants. In some cases, clinicogenomic information may be useful to manage the health status of other family members and other close relatives.

## Results

### Terminology Standards

In order to integrate structured genotype and phenotype data into any system, the first requirement is to determine data components, terminology standards, and identifiers of clinicogenomic information, for example, genotype data and its associated clinical interpretation.

In genomic terminology, the Human Gene Nomenclature Committee standardizes identifying gene symbols, identifiers, and variant nomenclature defined by the Human Genome Variation Society [6]. Reference SNP number (rs number) and reference SNP identifier (rsID) are used to identify every single SNP entry in the Single Nucleotide Polymorphism Database (dbSNP), which is the largest database maintained by the National Center for Biotechnology Information (NCBI). The dbSNP is interconnected with many other resources, for example, Entrez Gene, GenBank, the Universal Protein Resource, the International HapMap Project, the Pharmacogenomics Knowledge Base (PharmGKB), and the AlzGene, PDGene, SzGene databases through the rsID [33]. Additionally, in many types of personal genomic file formats (eg, 23andMe, deCODEme, and Navigenics), SNPs are identified by rsID.

DNA is a double stranded stretch, and every nucleated somatic cell has 22 pairs of autosomal, and one pair of sex chromosomes. This means for autosomal chromosomes we have two versions of DNA strands inherited via maternal and paternal sex cells. Different forms or variants of a particular polymorphism are called alleles. Because different alleles may have different degrees and types of clinical impact, rsID is insufficient alone to identify the clinicogenomic significance of SNPs. To have a heterozygote allele may not change the risk for the disease, but homozygote allele of the same SNP variant may change the risk for a disease dramatically. For example, in a study, the odds ratio for rs3218536 (A;G) was 0.8 (CI 0.7-1.0), and for the rs3218536 (A;A) 0.3 (0.1-0.9) [34]. Consequently, to identify clinically relevant SNPs, we need to use a combination of rsID and allele data as the minimum requirements.

DNA has a double strand (plus and minus or forward and reverse stands respectively), and every SNP can be identified using either of the two DNA strands. In various publications, the same alleles of SNPs are defined differently based on the orientation discrepancy [35]. Due to the double-stranded structure of DNA, both approaches are correct, but it is required to declare and use a standard.

Integration of variant data and clinical relevancies bring out the issue of terminological standardization. Unfortunately, conventional health information terminologies do not successfully support the genetic diseases. There is a critical gap between the databases, which involve many terms defining the genetic diseases, and the Systematized Nomenclature of Medicine (SNOMED) [36]. In order to address the chasm between medical vocabularies and bioinformatics resources, the clinical bioinformatics ontology (CBO) was developed and implemented. The CBO is a curated semantic network trying to combine a variety of clinical vocabularies, for example, SNOMED-Clinical Term (CT), Logical Observation Identifiers Names and Codes (LOINC), and NCBI bioinformatics resources [37,38].

In addition, the International Classification of Diseases (ICD) codes, which is also implemented in Turkey, is also preferred for identifying clinical conditions, but the released versions of ICD do not fully support genomic medicine [36]. Existing ICD versions are not efficient to manage all of the levels of clinical, pathologic, and genetic heterogeneities. It is expected that these will be managed in the next version, for example, ICD-11. The ICD-11, which is scheduled for release in 2015, is expected to be interoperable with other medical terminologies such as SNOMED-CT [39]. Nevertheless, it is an unavoidable requirement to develop a new taxonomy of diseases that will be based on information commons and knowledge networks, including a combination of molecular, social, environmental, and clinical data and health outcomes [40].

As explained in the next section, in the clinicogenomic knowledge base, the assessment of both evidence quality of study and effect size of these associations are critical for the analysis of the published results for clinicogenomic associations [41-47]. Despite emerged approaches and initiatives, standardized definitions and value assignment approaches are needed to categorize and use these associations in a consistent way.

Especially for polygenic complex diseases, impact degrees of clinicogenomic association may be different according to race, ethnicity, and environmental factors [48]. The terms of “ethnicity” and “race” refer to a sociocultural construct affecting both biological and environmental factors, and we need a general standard to categorize these terms.

Various predictive models, in clinical settings, may be useful to assess personal disease risk using relevant SNPs, for example, cumulative models, polygenic risk scores, etc. On the other hand, only a small number of holistic enviro-genomic models are available. Because most of the complex diseases are progressing as the interaction of genomic and environmental factors, it seems that, more enviro-genomic models will be produced in near future. Naturally, with the increase of the number and the value of predictive clinicogenomic models, we will need standardized definition and sharing methods for these models.

### Interoperability Standards

Health Level 7 (HL7) is a global organization developing health information standards. As an interoperability standard, the HL7 version 2.x (HL7 v2.x) is the most widely used all over the world. HL7 v2.x does not have not a clear information model, and contains many optional data fields. To overcome this vagueness problem, HL7 version 3 (HL7 v3) has been developed, which is based on an object oriented data model called Reference Information Model (RIM) [49]. HL7 v3 Clinical Document Architecture (CDA) is a document markup standard. A HL7 CDA document is produced to exchange information as part of the HL7 v3 standards, and aim to specify the structural and semantic aspects of clinical documents [50].

The HL7 Clinical Genomics (CG) Work Group (WG) develops standards intended to regulate interoperability issues in genomic medicine. *HL7 Version 2 Implementation Guide: Clinical Genomics; Fully LOINC-Qualified Genetic Variation Model* is based on both the *HL7 Version 2 Implementation Guide Laboratory Result Reporting to the EHR*, and the *HL7 Version 3 Genetic Variation* data model. This guide covers the reporting of the test results for sequencing and genotyping tests, and includes testing for DNA variants associated with diseases and pharmacogenomic applications [36,51,52]. *HL7 Version 2 Implementation Guide: Clinical Genomics; Fully LOINC-Qualified Genetic Variation Model* was the first example used by The Partners HealthCare Center for Personalized Genetic Medicine and the Intermountain Healthcare Clinical Genetics Institute to gather genetic test results and transmit them to a patient's EHR [51,52]. GeneInsight Suite (GeneInsight Lab, GeneInsight Clinic, and GeneInsight Network) is also a platform where clinical variant data sharing was based on HL7 standards [26,29,53,54].

The HL7 v3 genetic variation specification is based on the HL7 RIM. It uses the HL7 data types, vocabulary binding mechanisms built into the RIM and Bioinformatic Sequence Markup Language to model the sequence information. The root class in the genetic variation model is “genetic loci”, which describes a set of loci, such as a haplotype, a genetic profile, and genetic testing results of multiple variations or gene expression panels. The genetic loci model uses the genetic locus as an information unit to describe each of these loci. A genetic locus is composed of one or more individual alleles, sequences, and observed sequence variations and represents a single gene or coding region. Within this model, HL7 suggests the sharing of the essential part of raw genomic data via “encapsulation”, and extracting clinically relevant data via “bubble-up” based on a genomic decision support application [55].

HL7 CG-WG develops a CDA implementation guide (ie, Implementation Guide for CDA Release 2 Genetic Testing Report) to ensure the transmission of genetic testing reports using HL7 v3 RIM, and is appropriate for the level of granularity of human-readable reports [56].

### Clinicogenomic Knowledge Bases

Clinicians cannot extract clinical interpretation of variants directly from the medical sources due to temporal and cognitive limitations [57,58]. So, instead of incorporating all sequence data, integration of the clinical interpretations of variant data into medical records will be more efficient for clinical decision making [54,59]. Therefore, clinically relevant variants must be selected and presented with their clinical meaning, for example, clinicogenomic associations, along with an action plan for clinicians. Since the Human Genome Project, researchers have been discovering new clinicogenomic associations continuously, and it is critical to reinterpret variants and integrate new clinical interpretations into clinical processes [26].

Clinicogenomic associations, which are acquired via studies based on a candidate gene investigation or agnostic screening of complete genome, are published in the scientific literature [41]. Some clinicogenomic knowledge bases collect, curate, interpret, and categorize these published associations between genomic variations and clinical conditions. The Cancer Genome-wide Association and Meta Analyses Database is a part of Cancer Genomic Evidence-based Medicine Knowledge Base, and provides genome-wide association studies (GWAS), research, and meta-analysis about clinicogenomic associations [60,61]. ClinVar provides reports for variations and related phenotypes with evidences [62]. AlzGene [63], PDGene [64], and SzGene [65] are resources, which include manually curated PubMed articles, using systematic methods for Alzheimer’s disease, Parkinson’s disease, and schizophrenia, respectively. SNPedia is a wiki resource of human genetic variation as published in peer-reviewed research [66]. PharmGKB is a knowledge source containing clinically relevant genotype-phenotype and gene-drug relationships [67].

However, many of the existing knowledge bases for the clinical interpretation of variant data have different conventions. Also, they are not error proof and are not sustainable due to funding issues [54]. Especially for polygenic complex diseases, the impact degrees of clinicogenomic association may be different according to race, ethnicity, and environmental factors [48]. Therefore, in personalized risk assessment, it will be an ideal approach to use population specific clinicogenomic results, or at least findings from similar communities. If these are not possible, it might be conceivable to use other scientific resources with a confidence range. Experts have been advocating for the generation of centrally curated national repositories of clinically significant variants for the interpretation of an individual's genomic information, eventually [58,68]. To develop a national level clinicogenomic knowledge base is critical to consider consistency of clinicogenomic associations with the sociodemographic characteristics of citizens, and overcome the issues about sustainability.

Regarding published results of clinicogenomic associations, two major points are significant, evidence quality of study and effect size of these associations [41,42]. To measure the magnitude of impact for clinicogenomic associations, researchers usually prefer to use conventional approaches, for example, odds ratio (OR) and relative risks for case control studies and cohort studies, respectively. These values are presented with CI [43].

In GWAS, many defects and biases might be present based on study design, genotyping, or collected data quality that will affect the clinical value of results [41,44,45]. The quality of evidence is scored based on the type of study and how well the study is conducted [46], and some guidelines are proposed to calculate the evidence degree [47].

Human Genome Epidemiology Network has published the interim Venice guidelines to grade the cumulative evidence in genetic associations. This guideline is based on three criteria: (1) the amount of evidence (sample size), (2) replication of studies (determining association in different studies), and (3) protection from bias (Table 1). After the evaluation of a study, all considerations are categorized as A, B, and C, and finally, merged as a composite assessment using a semiquantitative index as strong, moderate, and weak epidemiological credibility for genetic associations [47].

**Table 1:** Venice interim guideline criteria for assessment of cumulative evidence on genetic associations [47].

Venice interim guideline criteria	Categories
Amount of evidence	Category A, sample size >1000 Category B, sample size >100 and <1000, Category C, sample size <100 (total number in cases and controls assuming 1:1 ratio)
Extent of replication	Category A, extensive replication including at least one well conducted meta-analysis with little between-study inconsistency. Category B, well conducted meta-analysis with some methodological limitations or moderate between-study inconsistency. Category C, no association; no independent replication; failed replication; scattered studies; flawed meta-analysis; or large inconsistency.
Protection from bias	Category A, bias, if at all present, could affect the magnitude, but probably not the presence of the association. Category B, no obvious bias that may affect the presence of the association, but there is considerable missing information on the generation of evidence. Category C, considerable potential for, or demonstrable bias, which can affect even the presence or absence of the association.

### Defining Clinical Significance

Today, Venice criteria are used to assess genomic association studies in several controlled and structured knowledge bases, for example, Alz-Gene, PD-Gene, and SZ-Gene [63-65]. For the importance of clinicogenomic association, some of the knowledge sources include additional data fields that define the magnitude of clinical effects and strength of the relationship between variants and diseases. In ClinVar, clinical significance is defined as a combination of impact and clinical function (eg, benign, pathogenic, protective, drug response, etc), and evidence for clinical significance is categorized regarding study count and type, such as in vitro studies, animal models, etc [62]. In the PharmGKB, a systematic categorization for evidence quality of clinicogenomic associations is extracted depending on methods and results of references [67], but impact value is not emphasized as a parameter. In SNPedia, magnitude is constructed as a subjective measure of interest for magnitude of impact and repute (good, bad) for quality of evidence, but these concepts are not well established. In GET-Evidence, clinicogenomic references are categorized according to their evidence degree (high, moderate, or low), and clinical significance (high, medium, or low) is used to produce impact score [69].

### Clinicogenomic Decision Support

The volume of variation data integrated into clinical practice exceeds the boundaries of unsupported human cognition and interpretive capacity. Additionally, the rapidly growing literature on clinicogenomic associations makes it more complicated to stay current for even experienced professionals [29]. Also, it is not reasonable to expect the interpretation of all clinicogenomic data by the limited number of genetics experts; we need more automated solutions to overcome these obstacles [70]. With the growing data load in the genomic era, in order to make informed decisions in a timely manner, the health care systems need to shift from expert-based practice to systems-supported practice [71].

Although there is a limited number of counter examples, in general, the clinical effect of a single SNP is minor (OR <2.00) [72,73]. Nevertheless, listing of clinicogenomic associations and their effects may be useful to report a limited number of independent associations. This is especially true for disease-associated SNPs with strong impact and strong evidence; users can share these one by one. At this point, using carefully chosen graphics and visualization techniques will be an efficient way of doing so. Various DTC genomic companies report personal genomic risk for various clinical conditions using graphics containing personal estimations [74].

Although the simplest way of reporting SNP variations is displaying these numerous variations in laboratory reports, it is clear that clinicians cannot interpret or evaluate this information stack. Modest value of clinicogenomic associations does not mean negligible, and some researchers try to develop polygenic risk models or panels assigning values for various SNP alleles, and calculate the total risk of disease for more effective risk prediction [75]. In the literature, several cumulative prediction models have been proposed, but most of these are criticized regarding comprehensive evaluation, especially for clinical utility [76].

SNPs could be used to produce a “genomic profile” for disease risk prediction, testing hundreds of thousands of loci across the personal genome. Today, most of the SNP-based risk assessment models have limited predictive utility and discriminative accuracy because most of the disease associated SNPs have small impacts [77,78]. It has been suggested that genomic risk scores based on large numbers of SNPs could explain more about the heritability than models based on a small number and rigorously validated SNPs. But there is a requirement to process large datasets to build such discriminative risk assessment models [79,80].

The genetic architecture of a disease refers to the number, effect size, genetic mode of action (additive, dominant, and/or epistatic), and allelic frequencies of the genetic polymorphisms. The prediction of genetic risk depends on the underlying genetic architecture. Indeed, the SNPs do not have to be the causative mutations. They just need to be in high linkage disequilibrium with the causative mutations so that there is a consistent association between the SNP and disease risk [81].

Different types of polygenic prediction models have been developed to combine the impact of disease associated SNP data, for example, count method, log odds method, multiplicative model, etc. The count method is the calculation of the total count of independent genomic risk alleles. The log odds method sums together the natural logarithm of the allelic OR for each risk allele [78]. DTC testing companies typically employ a multiplicative model to calculate lifetime risk in the absence of an established method for combining SNP risk estimates, for example, multiplication of ORs of each genotype and average population risk [82].

There are various cumulative models combining the impact of several clinicogenomic associations using arithmetic operators. In recessive models, only homozygote alleles are involved in the models, but in dominant models heterozygote SNPs are also a part of the cumulative models. Both in dominant and recessive models, the values of risk SNPs are accepted as one unit of impact. Models involving alterations of SNPs’ impact value regarding homozygote and heterozygote alleles are defined as an additive model [35,43].

Some of the models involve additional criteria, for example, family history [83]. But structured family history is not a mandatory part of EHR, and because of its dynamic characteristics, it is reasonable to collect and trace it at each visit from patients. It is clear that, similar to clinicogenomic associations, collection and reinterpretation of family history is critical to capture effective results with this type of predictive models.

Actually, genomic and environmental factors are involved in various degrees with the molecular etiology of diseases. In monogenetic diseases (eg, Huntington’s disease, phenylketonuria, hereditary cancer forms, etc), single gene mutations are predominantly the main cause of diseases. The genetic origins of the complex multifactorial diseases are much more complicated than the monogenetic diseases, which are a result of the complicated interactions between genetic and environmental causes [84].

Genomic information has lifelong value and one’s genomic findings can reveal others within families [23]. If a patient is found to have a disease associated variant, possibly other blood relatives would carry the similar risk, and the patient's health care provider could utilize this new clinical information [26]. This is especially important, not only because of the medical perspective, but also for security and privacy issues.

## Discussion

In this part of the miniseries, we have reviewed the scientific literature to extract the requirements for SNP data integrated into EMRs/EHRs.

In order to integrate structured genotype and phenotype data into any system, the first requirement is to determine data components, terminology standards, and identifiers of clinicogenomic information, for example, genotype data and its associated clinical interpretation. Also, we need interoperability standards such as HL7 v2 or v3 to share information between stakeholders.

Because of the huge amount of clinically relevant genomic data and fast translation of this information to a clinical domain, we need clinical decision support capability. To ensure this capability, we also need a continuously updated accredited and structured knowledge base, and assessment approaches to interpret these genomic variations.

In the next part of the miniseries, we will present our study to extend capabilities of NHIS-T to handle SNP data, and its clinical interpretation to assess personal disease risk, and propose possible solutions regarding these requirements.

## Abbreviations

CBO: clinical bioinformatics ontology
CDA: Clinical Document Architecture
dbSNP: Single Nucleotide Polymorphism Database
DNA: deoxyribonucleic acid
DTC: direct-to-consumer
EHR: electronic health record
EMR: electronic medical record
GWAS: genome-wide association studies
HL7: Health Level 7
HL7 v2.x: HL7 version 2.x
HL7 v3: HL7 version 3
HL7 CG: Health Level 7 Clinical Genomic
ICD: International Classification of Diseases
LOINC: Logical Observation Identifiers Names and Codes
NCBI: National Center for Biotechnology Information
NGS: next generation sequencing
NHIS-T: National Health Information System of Turkey
OR: odds ratio
PharmGKB: Pharmacogenomics Knowledge Base
RIM: Reference Information Model
rsID: reference SNP identifier
rs number: reference SNP number
SNOMED: Systematized Nomenclature of Medicine
SNOMED-CT: Systematized Nomenclature of Medicine Clinical Term
SNP: single nucleotide polymorphism
WES: whole exome sequencing
WG: Work Group
WGS: whole genome sequencing
